# Symptom normalization, care-seeking delay, and health-related quality of life in adults with gastroesophageal reflux disease: a cross-sectional study

**DOI:** 10.3389/fmed.2026.1969209

**Published:** 2026-10-14

**Authors:** Eman Shorog, Geetha Kandasamy, Asma M. Alshahrani, Leen Alshahrani, Wajd Fahad Al.qahtani, Walaa Abu Rukbah, Shadin Nasreldin Suliman, Bashayr Alsuwayni, Vasudevan Mani, Abdalla Ali Abdalla

**Affiliations:** 1 Department of Clinical Pharmacy, College of Pharmacy, King Khalid University, Abha, Saudi Arabia; 2 Department of Clinical Pharmacy, College of Pharmacy, Shaqra University, Dawadimi, Saudi Arabia; 3 Department of Public Health, College of Applied Medical Sciences, King Khalid University, Abha, Saudi Arabia; 4 Department of Pharmacy Practice, Faculty of Pharmacy, University of Tabuk, Tabuk, Saudi Arabia; 5 King Saud University Medical City, Riyadh, Saudi Arabia; 6 Department of Pharmacology and Toxicology, College of Pharmacy, Qassim University, Buraydah, Saudi Arabia; 7 Department of Paediatrics, Faculty of Medicine, University of Tabuk, Tabuk, Saudi Arabia

**Keywords:** care-seeking delay, gastroesophageal reflux disease (GERD), GERD-HRQoL, GerdQ, health-related quality of life, symptom normalization

## Abstract

**Background:**

Gastroesophageal reflux disease (GERD) is associated with significant physical, psychological, and functional impairment. Although patients frequently normalize reflux symptoms and postpone medical consultation, the extent to which these behaviors influence symptom burden and health-related quality of life (HRQoL) remains unclear. This study evaluated the associations between prespecified Symptom Normalization and Care-Seeking Delay conceptual domain scores, GERD symptom severity, and HRQoL in adults with GERD.

**Methods:**

A cross-sectional study was conducted among 218 adults with physician-established diagnosis of GERD attending King Khalid University Medical City and affiliated primary healthcare centers in Abha, Saudi Arabia. Data were collected using Gastroesophageal Reflux Disease Questionnaire (GerdQ), GERD-HRQoL, and prespecified conceptual domain scores for symptom normalization and care-seeking delay, and analyzed using correlation and regression methods.

**Results:**

A total of 218 participants were included, with the majority being female (87.6%) and aged 18–40 years (49.0%). Among participants with physician-established GERD, 33.5% had GerdQ scores ≥8, indicating higher symptom burden. GERD symptom severity showed a strong positive correlation with GERD-HRQoL scores (*ρ* = 0.704, *p* < 0.001), indicating poorer quality of life with greater symptom burden. The prespecified Symptom Normalization and Care-Seeking Delay conceptual domain scores were strongly correlated with each other (*ρ* = 0.734, *p* < 0.001), while both showed weak correlations with GERD symptom severity. In multivariable linear regression, GerdQ score was the only variable significantly associated with GERD-HRQoL (B = 0.971, 95% CI: 0.808–1.134, *p* < 0.001), and the model explained 50.6% of the variance in GERD-HRQoL scores (R^2^ = 0.506; adjusted R^2^ = 0.472). The prespecified Symptom Normalization and Care-Seeking Delay conceptual domain scores were not statistically significantly associated with GERD-HRQoL after adjustment.

**Conclusion:**

Among adults with physician-established GERD, greater current symptom burden was independently associated with poorer HRQoL, while higher Symptom Normalization conceptual domain scores were strongly associated with higher Care-Seeking Delay conceptual domain scores. These findings suggest that symptom appraisal and healthcare-seeking behaviors should be considered alongside symptom severity when evaluating the overall burden of GERD.

## 1 Introduction

A repeated retrograde movement of stomach contents into the oesophagus and occasionally the oropharynx is the hallmark of gastroesophageal reflux disease (GERD), a chronic gastrointestinal condition. Esophageal reflux is typically associated with disturbances in gastrointestinal motility and either structural defects that compromise the barrier function of the esophagogastric junction (EGJ) or intrinsic functional dysfunction (1). Heartburn, regurgitation, and chest pain are commonly reported GERD symptoms, while atypical or extra-esophageal manifestations may include hoarseness, persistent cough, and globus sensation (1, 2). These symptoms may interfere with everyday activities such as sleeping, eating, and working and are associated with reduced quality of life (QoL), an important outcome measure in GERD management (3).

GERD is common worldwide, although prevalence estimates vary across populations. GERD symptoms have been reported in 45%–71% of patients with asthma (4–6), and epidemiological studies indicate that GERD is increasingly recognized globally (7, 8). Approximately 15%–25% of the global population is estimated to have GERD (9), while reported prevalence in Saudi Arabia ranges from 15% to 45.4% (10–12). Complications such as erosive oesophagitis, Barrett's oesophagus, and esophageal cancer may occur in more severe disease (13). Prevalence estimates are approximately 10%–20% in Western countries and generally lower, although increasing, in parts of Asia and Africa (2). A global analysis reported an overall GERD prevalence of 13.98%, with the Middle East among the regions reporting higher prevalence estimates (14). Higher incidence has also been reported among smokers, individuals with obesity, and those over 50 years of age (2), while some studies have reported a higher risk among males than females (15).

Genetic factors may contribute to GERD susceptibility, while obesity and lifestyle factors such as smoking and physical inactivity have also been associated with the condition (16, 17). GERD symptoms are associated with impaired daily functioning and health-related quality of life (HRQoL) (18, 19), and studies have described relationships between symptom burden, daily functioning, and HRQoL (20–22). However, differences may exist between patients’ experiences and physicians’ perceptions of symptom severity and management needs, particularly regarding lifestyle modification (23). Such differences highlight the importance of considering patient perspectives when examining symptom normalization, care-seeking behavior, and HRQoL (24, 25). Patients may also use self-management strategies, while adherence to proton pump inhibitors (PPIs) and lifestyle modifications may vary according to patient understanding, motivation, and feasibility (26). Research further indicates differences between patients’ reported symptom burden and clinicians’ perceptions of symptom severity and daily disruption (27).

Care-seeking behavior is another important aspect of the patient experience. Physicians report that some patients delay consultation because symptoms are perceived as minor or are initially managed independently, with medical consultation more often occurring when symptoms become frequent or interfere with daily activities (28). Greater GERD symptom severity has been associated with poorer HRQoL, with moderate-to-severe heartburn and regurgitation linked to greater impairment in daily functioning and well-being (29). Similarly, the SILC epidemiological study in China reported that even mild reflux symptoms may be associated with impaired daily functioning and QoL (30). Evidence from Palestine has also demonstrated associations between GERD symptom severity and psychosocial health, including stress, anxiety, and daily functioning (31).

Despite the established relationship between GERD symptom burden and HRQoL, relatively limited attention has been given to symptom normalization and care-seeking delay in the Saudi clinical context. Examining these patient-reported dimensions may provide a more comprehensive description of the GERD experience and identify areas for further investigation. Therefore, this study aimed to examine the associations among GERD symptom burden, the prespecified Symptom Normalization and Care-Seeking Delay conceptual domain scores, and HRQoL among adults with physician-established GERD attending outpatient healthcare settings in the Aseer region of Saudi Arabia.

## 2 Methods

### 2.1 Study design and setting

A descriptive, cross-sectional study was conducted among adult patients with physician-diagnosed GERD attending the Gastroenterology Outpatient Department at King Khalid University Medical City (KKUMC), Abha, Saudi Arabia, and affiliated Primary Health Care (PHC) centers. Recruitment was carried out during routine outpatient visits between December 2025 and March 2026. Eligible participants were identified by the attending physicians according to predefined inclusion and exclusion criteria. Participants had a physician-established diagnosis of GERD before enrolment and were recruited during routine outpatient visits. Data were collected using a structured patient-reported questionnaire assessing symptom severity, symptom normalization, care-seeking behaviour, and health-related quality of life. Written informed consent was obtained from all participants before enrolment.

### 2.2 Sample size and sampling method

The sample size was determined based on the feasibility of recruiting eligible patients during the study period. Consecutive sampling was used, whereby all eligible adult patients with physician-diagnosed GERD attending the participating outpatient clinics during the study period were invited to participate. A total of 218 participants were included in the final analysis.

### 2.3 Study population

Adult patients aged 18 years or older with a physician-established diagnosis of GERD were eligible to participate. The diagnosis was established by the treating physician based on the available clinical information, which could include clinical assessment, previous endoscopic findings, pH monitoring results, documented response to proton pump inhibitor therapy, or a previously documented GERD diagnosis in the medical record/problem list, as applicable. Both newly diagnosed individuals and those with a previous diagnosis of GERD were included, provided they had experienced reflux symptoms for at least three months. Participants were required to be able to understand and complete the study questionnaire independently or with minimal assistance and to provide written informed consent. Patients were excluded if they had a history of upper gastrointestinal malignancy or major upper gastrointestinal surgery, severe cognitive impairment or psychiatric illness that could compromise the reliability of self-reported data, pregnancy, or critical illness. Medication-related variables were not collected, as the primary objective of the study was to evaluate symptom perception, symptom normalization, care-seeking behavior, and health-related quality of life rather than treatment effectiveness or medication use.

### 2.4 Data collection procedure

Eligible patients were identified by the attending clinicians during routine outpatient visits based on the predefined inclusion and exclusion criteria. After completing their clinical consultation, patients who agreed to participate were provided with information about the study, and written informed consent was obtained before data collection. Participants were then provided with a Google Form link containing the structured questionnaire, which they completed using their personal electronic devices. Participants were encouraged to complete the questionnaire independently, and assistance was provided only when required to clarify questions without influencing their responses. The submitted responses were automatically recorded in a secure database and subsequently reviewed for completeness before statistical analysis.

### 2.5 Data collection tools

Data were collected using a structured questionnaire comprising three components.

#### 2.5.1 GERD symptom severity

The Gastroesophageal Reflux Disease Questionnaire (GerdQ), developed and validated by Jones et al. (32), is a validated six-item patient-reported instrument designed to evaluate the frequency of GERD-related symptoms experienced during the preceding seven days. The questionnaire includes four positive predictor items (heartburn, regurgitation, sleep disturbance due to heartburn and/or regurgitation, and use of additional medication for symptom relief) and two negative predictor items (epigastric pain and nausea). Each item is scored on a four-point scale according to symptom frequency during the previous week (0 days, 1 day, 2–3 days, and 4–7 days). The positive predictor items are scored in ascending order from 0 to 3 based on increasing symptom frequency, whereas the two negative predictor items are reverse scored (from 3 to 0), as they are less specific for GERD. The individual item scores are summed to obtain a total GerdQ score ranging from 0 to 18, with higher scores indicating greater current GERD symptom burden. In the original validation study by Jones et al., a total score of ≥8 was associated with a higher likelihood of GERD. In the present study, eligibility and classification of participants as having GERD were based on a prior physician-established diagnosis, rather than on the GerdQ score. The GerdQ score was used to characterize current symptom burden rather than to establish or exclude the diagnosis of GERD. Therefore, the ≥8 threshold was used only as a descriptive threshold for higher symptom burden and was not interpreted as a diagnostic cutoff.

#### 2.5.2 Quality of life

The Gastroesophageal Reflux Disease Health-Related Quality of Life (GERD-HRQL) questionnaire, developed and validated by Velanovich (33), is a disease-specific, patient-reported outcome measure designed to assess the severity of GERD symptoms and their impact on health-related quality of life. The original short version consists of 10 symptom-specific items that evaluate heartburn-related symptoms, dysphagia, odynophagia, the impact of GERD on eating and sleeping, medication use, and overall satisfaction with symptom control. Each item is scored using a 6-point Likert scale ranging from 0 to 5, where 0 indicates the absence of symptoms, 1 indicates very mild symptoms, 2 indicates mild symptoms, 3 indicates moderate symptoms, 4 indicates severe symptoms, and 5 indicates very severe or incapacitating symptoms. All items are scored in the same direction, with no reverse scoring required. The individual item scores are summed to obtain a total GERD-HRQoL score ranging from 0 to 50, with higher scores indicating greater symptom severity and poorer GERD-related health-related quality of life, whereas lower scores indicate better symptom control and improved quality of life. The GERD-HRQoL is primarily used to quantify symptom burden and assess treatment outcomes and does not have a universally accepted diagnostic cutoff value.

#### 2.5.3 Symptom normalization and care-seeking delay

Symptom perception and care-seeking behavior were assessed using a brief, purpose-designed eight-item questionnaire. The initial items were developed based on the study objectives and relevant literature addressing symptom normalization, self-management, and healthcare-seeking behavior. Before data collection, the items were organized *a priori* into two conceptual domains: Symptom Normalization and Care-Seeking Delay, based on their conceptual relevance to the respective domains. For content validity, the initial items were reviewed by an expert panel with relevant clinical and research expertise to assess their relevance, clarity, comprehensiveness, and appropriateness. Feedback from the experts was incorporated to refine the wording and content of the items.

For linguistic validity, the questionnaire was translated from English into Arabic using a forward-translation and back-translation process to ensure linguistic and conceptual equivalence between the two versions. The translated version was reviewed for clarity, cultural appropriateness, and consistency of meaning, and discrepancies identified during the translation process were resolved before administration. The two prespecified conceptual domains comprised four items each. Symptom Normalization reflected the extent to which patients perceived or tolerated GERD symptoms as a routine or insufficiently serious part of life, whereas Care-Seeking Delay reflected the timing and reasons for delaying medical consultation. Responses were rated on a five-point Likert scale, with higher scores indicating greater symptom normalization and longer delays in seeking medical care.

#### 2.5.4 Pilot testing and refinement of the questionnaire

Prior to the main study, the complete study questionnaire was pilot tested among 25 adult patients with GERD to assess its clarity, comprehensibility, feasibility, and average completion time. Feedback obtained from participants was reviewed, and minor modifications were made to improve the wording and clarity of selected items without altering the intended meaning or scoring system of the validated instruments. Data obtained from the pilot study were not included in the final analysis.

### 2.6 Reliability and exploratory factor structure of study questionnaires

The internal consistency of the study questionnaires was evaluated using Cronbach's alpha coefficient. The GerdQ demonstrated good internal consistency (Cronbach's *α* = 0.85), while the GERD-HRQoL questionnaire demonstrated acceptable internal consistency (Cronbach's *α* = 0.76). The purpose-designed Symptom Normalization and Care-Seeking Delay domain scores also demonstrated good internal consistency, with Cronbach's alpha values of 0.866 and 0.914, respectively.

The factor structure of the eight items comprising the purpose-designed Symptom Normalization and Care-Seeking Delay conceptual domains was explored using exploratory factor analysis (EFA). The Kaiser–Meyer–Olkin (KMO) measure was 0.901, and Bartlett's test of sphericity was statistically significant (*χ*^2^ = 1333.614, df = 28, *p* < 0.001), indicating that the data were suitable for factor analysis. Principal axis factoring was used, and factor retention was assessed using eigenvalues, the scree plot, and parallel analysis. A one-factor solution was supported, with an eigenvalue of 5.332 and 66.65% of the total variance explained. Factor loadings ranged from 0.602 to 0.872. No rotation was applied because only one factor was retained. The EFA supported a common underlying factor across the eight items but did not establish discriminant validity between the two prespecified conceptual domains. Accordingly, the four Symptom Normalization items and four Care-Seeking Delay items were retained as prespecified conceptual domain scores rather than as empirically distinct factors. These domain scores were examined separately in subsequent analyses, consistent with the *a priori* study framework.

### 2.7 Statistical analysis

Data analysis was conducted using IBM SPSS Statistics for Windows, Version 27.0 (IBM Corp., Armonk, NY, USA). Categorical data were expressed as frequencies and percentages, whereas continuous variables were summarized as median [interquartile range (IQR)]. The distribution of continuous variables was evaluated using the Kolmogorov–Smirnov and Shapiro–Wilk tests. Since all continuous variables demonstrated non-normal distributions (*p* < 0.05), non-parametric methods were applied for comparative analyses. The Mann–Whitney U test was used to compare questionnaire scores between two independent groups, while the Kruskal–Wallis test was applied for comparisons involving more than two groups.

Associations between GerdQ, GERD-HRQoL, and the prespecified Symptom Normalization and Care-Seeking Delay conceptual domain scores were examined using Spearman's rank correlation analysis. Covariates for the multivariable linear regression model were selected *a priori* based on clinical relevance and evidence from the existing literature. Age group, gender, educational level, BMI category, and smoking status were included as categorical covariates and entered into the regression model using dummy coding, with 18–40 years, male, graduate education, normal BMI, and non-smoking used as the respective reference categories. GerdQ and the prespecified Symptom Normalization and Care-Seeking Delay conceptual domain scores were included as continuous variables based on the study objectives Multivariable linear regression was performed to examine factors associated with GERD-HRQoL scores. Although the outcome variable was not normally distributed, linear regression was considered appropriate because the assumptions of linear regression primarily relate to the distribution of residuals rather than the dependent variable itself. Model assumptions, including multicollinearity, were evaluated using tolerance and variance inflation factor (VIF) values before interpretation of the regression results. Regression diagnostics were evaluated before interpretation of the model. Linearity was assessed by visual inspection of the standardized residual-versus-predicted-value plot, which showed no marked nonlinear pattern. The residual distribution was assessed using a histogram and normal P–P plot; residuals were approximately centered around zero, although some departures from normality were observed in the tails. Potential influential observations and outliers were assessed using standardized residuals, leverage values, and Cook's distance; the maximum Cook's distance was 0.315, with no observation identified as having undue influence on the fitted model. Heteroscedasticity was assessed by visual inspection of the residual-versus-predicted-value plot and by an auxiliary regression of the squared unstandardized residuals on the full set of model predictors. The auxiliary regression was statistically significant [F(14, 203) = 2.317, *p* = 0.006], indicating evidence of heteroscedasticity. Therefore, heteroscedasticity-robust inference was obtained using the generalized linear model (GENLIN) procedure, specifying a normal distribution with an identity link and the robust Huber–White (sandwich) covariance estimator. SPSS GENLIN does not identify this robust covariance estimator using the HC0–HC3 classification; therefore, no specific HC variant was assigned. The regression coefficient estimates remained those of the corresponding ordinary least-squares model, while robust covariance estimates were used to obtain the standard errors, 95% confidence intervals, and *p*-values. Independence of residuals was assessed using the Durbin–Watson statistic, which was 1.958. Multicollinearity was assessed using tolerance and variance inflation factor (VIF) values, which ranged from 1.087 to 2.615. Exploratory factor analysis was performed using Principal Axis Factoring. Factor retention was assessed using eigenvalues, scree-plot inspection, and parallel analysis and internal consistency reliability was assessed using Cronbach's alpha coefficient. All statistical tests were two-sided, and a *p*-value < 0.05 was considered statistically significant.

### 2.8 Ethical considerations

The study protocol was reviewed and approved by the Institutional Ethics Committee of King Khalid University (Approval No. KKU-186-2025-31). Written informed consent was obtained from all participants prior to data collection. Participation was voluntary, and participants were informed of their right to withdraw from the study at any time without any consequences. Participant anonymity and confidentiality of all collected data were maintained throughout the study.

## 3 Results

### 3.1 Reliability and exploratory factor structure of study questionnaires

The study questionnaires demonstrated good internal consistency, with Cronbach's alpha values of 0.85 for GerdQ, 0.76 for GERD-HRQoL, 0.866 for the prespecified Symptom Normalization conceptual domain score, and 0.914 for the prespecified Care-Seeking Delay conceptual domain score. Exploratory factor analysis of the eight purpose-designed items demonstrated excellent sampling adequacy (KMO = 0.901), and Bartlett's test of sphericity was significant (*χ*^2^ = 1333.614, df = 28, *p* < 0.001). Principal axis factoring supported a one-factor solution, with an eigenvalue of 5.332 and 66.65% of the total variance explained. Factor loadings ranged from 0.602 to 0.872, and parallel analysis supported retention of one factor (Supplementary Table S2). Because only one factor was retained, no rotation was applied. The EFA supported a common underlying factor across the eight items but did not establish discriminant validity between the two prespecified conceptual domains. Accordingly, the four Symptom Normalization items and four Care-Seeking Delay items were retained as prespecified conceptual domain scores and examined separately in subsequent analyses in accordance with the *a priori* study framework. All continuous variables showed significant deviation from normality based on the Kolmogorov–Smirnov and Shapiro–Wilk tests (all *p* < 0.001). Accordingly, non-parametric tests were used for group comparisons and correlation analyses, as appropriate (Supplementary Table S3).

The sociodemographic and clinical characteristics of the study participants are presented in Table 1. A total of 218 participants were included in the study. Nearly half of the participants (49.0%, *n* = 107) were aged 18–40 years, while 23.9% (*n* = 52) were older than 60 years, 20.2% (*n* = 44) were aged 41–50 years, and 6.9% (*n* = 15) were aged 51–60 years. The majority of participants were female (87.6%, *n* = 191), whereas 12.4% (*n* = 27) were male. More than half of the participants were married (58.3%, *n* = 127), while 41.7% (*n* = 91) were single or divorced. Regarding educational attainment, 60.6% (*n* = 132) were graduates, 33.5% (*n* = 73) had completed secondary education, 5.0% (*n* = 11) were postgraduates, and 0.9% (*n* = 2) had primary education. Most participants were unemployed or retired (70.6%, *n* = 154), whereas 29.4% (*n* = 64) were employed. Monthly household income was relatively evenly distributed, with 29.8% (*n* = 65) reporting an income of ≥15,000 SAR, followed by 24.8% (*n* = 54) earning 10,000–14,999 SAR, 23.9% (*n* = 52) earning 5,000–9,999 SAR, and 21.6% (*n* = 47) earning <5,000 SAR. Most participants resided in urban areas (92.2%, *n* = 201), while only 7.8% (*n* = 17) lived in rural areas. Approximately one-third (33.5%, *n* = 73) reported having health insurance, whereas 66.5% (*n* = 145) did not. Based on BMI classification, the majority of participants had normal body weight (57.8%, *n* = 126), followed by overweight (28.0%, *n* = 61), obese (8.3%, *n* = 18), and underweight (6.0%, *n* = 13). Most participants were non-smokers (95.0%, *n* = 207), with only 5.0% (*n* = 11) reporting current smoking.

**Table 1:** Sociodemographic and clinical characteristics of the study participants.

Variable	Category	n (%)
Age	18–40 years	107 (49%)
	41–50 years	44 (20.2%)
	51–60 years	15 (6.9%)
	More than 60 years	52 (23.9%)
Gender	Male	27 (12.4%)
	Female	191 (87.6%)
Marital status	Married	127 (58.3%)
	Single/Divorced	91 (41.7%)
Educational level	Primary	2 (0.9%)
	Secondary	73 (33.5%)
	Graduate	132 (60.6%)
	Postgraduate	11 (5.0%)
Occupation	Employed	64 (29.4%)
	Unemployed/Retired	154 (70.6%)
Monthly income	<5000 SAR	47 (21.6%)
	5000–9999 SAR	52 (23.9%)
	10000–14999 SAR	54 (24.8%)
	≥15000 SAR	65 (29.8%)
Residence	Rural	17 (7.8%)
	Urban	201 (92.2%)
Health Insurance	Yes	73 (33.5%)
	No	145 (66.5%)
BMI	Underweight	13 (6.0%)
	Normal	126 (57.8%)
	Overweight	61 (28.0%)
	Obese	18 (8.3%)
Smoking	Yes	11 (5.0%)
	No	207 (95.0%)

Table 2 presents the distribution of responses to the GerdQ and GERD-HRQoL questionnaires. For the Gerd-Q, the majority of participants reported no heartburn (43.1%, *n* = 94) and no regurgitation (39.9%, *n* = 87) during the preceding week. Similarly, most respondents reported no upper stomach pain (35.3%, *n* = 77), no nausea (40.8%, *n* = 89), no use of over-the-counter (OTC) medication for reflux symptoms (61.0%, *n* = 133), and no sleep disturbance related to GERD (42.7%, *n* = 93). Nevertheless, a considerable proportion experienced symptoms on one or more days during the week, particularly for upper stomach pain (64.7%) and nausea (59.2%), indicating varying symptom frequency among participants. Based on the GerdQ score, 145 participants (66.5%) had a score <8, while 73 participants (33.5%) had a score ≥8, indicating a higher current symptom burden. Responses to the GERD-HRQoL questionnaire indicated that most participants reported no impairment (score = 0) across all quality-of-life domains. Specifically, 43.1% (*n* = 94) reported no heartburn severity, 45.9% (*n* = 100) reported no heartburn while lying down, 65.6% (*n* = 143) reported no heartburn while standing, and 54.1% (*n* = 118) experienced no heartburn after meals. Likewise, nearly half of the participants reported no difficulty swallowing (46.3%, *n* = 101) or pain while swallowing (50.0%, *n* = 109). More than half also reported no gassy or bloating sensation (51.8%, *n* = 113), no heartburn-related sleep disruption (56.4%, *n* = 123), no dietary modification due to GERD symptoms (45.4%, *n* = 99), and no impact of GERD medication on daily activities (55.0%, *n* = 120). Although the majority of participants selected the lowest severity category across GERD-HRQoL items, a notable proportion reported mild-to-moderate symptom-related impairment (scores 1–3), while relatively few participants reported severe impairment (scores 4–5), suggesting that the overall burden of GERD on health-related quality of life was generally low to moderate within the study population.

**Table 2:** Distribution of responses to the gerdQ and GERD-HRQoL questionnaires and overall gerdQ classification.

A. Gastroesophageal Reflux Disease Questionnaire (GerdQ)				
Items	0 days	1 day	2–3 days	4–7 days
Heartburn	94 (43.1%)	63 (28.9%)	40 (18.3%)	21 (9.6%)
Regurgitation	87 (39.9%)	71 (32.6%)	42 (19.3%)	18 (8.3%)
Upper stomach pain*	77 (35.3%)	53 (24.3%)	63 (28.9%)	25 (11.5%)
Nausea*	89 (40.8%)	60 (27.5%)	42 (19.3%)	27 (12.4%)
OTC medication use	133 (61.0%)	33 (15.1%)	26 (11.9%)	26 (11.9%)
Sleep disturbance	93 (42.7%)	66 (30.3%)	41 (18.8%)	18 (8.3%)

Overall GERD-Q Classification	N (%)
GERD-Q score <8 (Lower symptom burden)	145 (66.5%)
GERD-Q score ≥8 (Higher symptom burden)	73 (33.5%)

C. Gastroesophageal Reflux Disease Health-Related Quality of Life (GERD-HRQoL) Questionnaire						
Items	0	1	2	3	4	5
Heartburn severity	94 (43.1%)	51 (23.4%)	41 (18.8%)	27 (12.4%)	3 (1.4%)	2 (0.9%)
Heartburn when lying down	100 (45.9%)	55 (25.2%)	39 (17.9%)	17 (7.8%)	6 (2.8%)	1 (0.5%)
Heartburn when standing up	143 (65.6%)	45 (20.6%)	18 (8.3%)	9 (4.1%)	2 (0.9%)	1 (0.5%)
Heartburn after meals	118 (54.1%)	45 (20.6%)	30 (13.8%)	18 (8.3%)	3 (1.4%)	4 (1.8%)
Difficulty swallowing	101 (46.3%)	61 (28.0%)	30 (13.8%)	13 (6.0%)	11 (5.0%)	2 (0.9%)
Pain while swallowing	109 (50.0%)	57 (26.1%)	31 (14.2%)	15 (6.9%)	3 (1.4%)	3 (1.4%)
Gassy/ bloating feeling	113 (51.8%)	47 (21.6%)	27 (12.4%)	23 (10.6%)	3 (1.4%)	5 (2.3%)
Heartburn waking from sleep	123 (56.4%)	47 (21.6%)	26 (11.9%)	18 (8.3%)	2 (0.9%)	2 (0.9%)
Dietary modification due to symptoms	99 (45.4%)	66 (30.3%)	31 (14.2%)	16 (7.3%)	2 (0.9%)	4 (1.8%)
Medication effects on daily life	120 (55.0%)	40 (18.3%)	30 (13.8%)	18 (8.3%)	6 (2.8%)	4 (1.8%)

* Reverse-scored items

Table 3 summarizes participants’ responses to the prespecified Symptom Normalization and Care-Seeking Delay conceptual domain items. For the Symptom Normalization domain, more than half of the participants (54.1%, *n* = 118) disagreed or strongly disagreed that reflux symptoms were a normal part of life, whereas 19.3% (*n* = 42) agreed or strongly agreed with this statement. However, a substantial proportion acknowledged behaviors consistent with symptom normalization. Specifically, 36.7% (*n* = 80) agreed or strongly agreed that they ignored GERD symptoms instead of seeking medical care, 38.5% (*n* = 84) considered their symptoms insufficiently serious to warrant medical consultation, and 39.9% (*n* = 87) reported self-managing their symptoms without professional medical assistance. Neutral responses ranged from 17.9% to 26.6% across the four items, indicating uncertainty among a considerable proportion of participants. Regarding the Care-Seeking Delay domain, approximately one-fifth of participants (22.9%, *n* = 50) agreed or strongly agreed that they took a long time to seek professional care for their symptoms, while 45.8% (*n* = 100) disagreed or strongly disagreed with this statement. Nevertheless, delayed care behaviors were commonly reported for specific reasons. Nearly two-fifths of participants agreed or strongly agreed that they delayed seeking care because they expected symptoms to resolve on their own (39.4%, *n* = 86) or expected spontaneous improvement (41.2%, *n* = 90). Similarly, 34.4% (*n* = 75) agreed or strongly agreed that they postponed medical consultation because they were uncertain about the severity of their symptoms. Across all care-seeking delay items, approximately one-quarter to one-third of participants selected the neutral response, suggesting variability in healthcare-seeking attitudes.

**Table 3:** Distribution of responses to the prespecified symptom normalization and care-seeking delay conceptual domain items.

D. Symptom Normalization Conceptual Domain					
Item	Strongly Disagree	Disagree	Neutral	Agree	Strongly Agree
Perceives reflux symptoms as a normal part of life	80 (36.7)	38 (17.4%)	58 (26.6%)	37 (17.0%)	5 (2.3%)
Ignores GERD symptoms instead of seeking care	57 (26.1%)	38 (17.4%)	43 (19.7%)	71 (32.6%)	9 (4.1%)
Considers symptoms not serious enough for medical consultation	61 (28.0%)	32 (14.7%)	41 (18.8%)	73 (33.5%)	11 (5.0%)
Self-manages symptoms without medical help	62 (28.4%)	30 (13.8%)	39 (17.9%)	74 (33.9%)	13 (6.0%)

E. Care-Seeking Delay Conceptual Domain					
Item	Strongly Disagree	Disagree	Neutral	Agree	Strongly Agree
Took a long time to seek professional care	57 (26.1%)	43 (19.7%)	68 (31.2%)	40 (18.3%)	10 (4.6%)
Delayed care expecting symptoms to resolve	46 (21.1%)	36 (16.5%)	50 (22.9%)	75 (34.4%)	11 (5.0%)
Postponed care due to uncertainty about severity	62 (28.4%)	31 (14.2%)	50 (22.9%)	67 (30.7%)	8 (3.7%)
Delayed care expecting spontaneous improvement	53 (24.3%)	25 (11.5%)	50 (22.9%)	79 (36.2%)	11 (5.0%)

Table 4 presents the associations between participant characteristics and the median (interquartile range) scores for GerdQ, GERD-HRQoL, and the prespecified Symptom Normalization and Care-Seeking Delay conceptual domain scores. Overall, few sociodemographic and clinical characteristics were significantly associated with the study outcomes. Since the score distributions were non-normally distributed, non-parametric tests (Kruskal–Wallis and Mann–Whitney U tests) were applied. Age group showed a statistically significant association with GERD-HRQoL scores (*p* = 0.044), with participants aged more than 60 years demonstrating higher GERD-HRQoL scores [median (IQR): 11 (8)] compared with younger age groups. However, no significant differences were observed across age groups for GerdQ scores (*p* = 0.118), Symptom Normalization conceptual domain scores (*p* = 0.515), or Care-Seeking Delay conceptual domain scores (*p* = 0.505). No significant association was observed between gender and any of the assessed scores. GerdQ (*p* = 0.533), GERD-HRQoL (*p* = 0.813), Symptom Normalization conceptual domain scores (*p* = 0.466), and Care-Seeking Delay conceptual domain scores (*p* = 0.515) were comparable between male and female participants. Educational level was significantly associated with Care-Seeking Delay conceptual domain scores (*p* = 0.010). Participants with postgraduate education showed higher Care-Seeking Delay conceptual domain scores [median (IQR): 14 (4)] compared with those with lower educational attainment. Symptom Normalization conceptual domain scores showed a borderline association with education level (*p* = 0.054). However, GerdQ (*p* = 0.183) and GERD-HRQoL (*p* = 0.256) scores did not differ significantly across educational categories. BMI categories were not significantly associated with GerdQ (*p* = 0.459), GERD-HRQoL (*p* = 0.895), Symptom Normalization conceptual domain scores (*p* = 0.476), or Care-Seeking Delay conceptual domain scores (*p* = 0.612). Similarly, smoking status showed no statistically significant differences in GerdQ (*p* = 0.667), GERD-HRQoL (*p* = 0.409), or Symptom Normalization conceptual domain scores (*p* = 0.390). Care-Seeking Delay conceptual domain scores among smokers showed a borderline trend toward higher values compared with non-smokers; however, this did not reach statistical significance (*p* = 0.067). Monthly family income was not significantly associated with GerdQ (*p* = 0.141), GERD-HRQoL (*p* = 0.293), Symptom Normalization conceptual domain scores (*p* = 0.259), or Care-Seeking Delay conceptual domain scores (*p* = 0.523). Similarly, residence status (rural versus urban) was not significantly associated with GerdQ (*p* = 0.695), GERD-HRQoL (*p* = 0.475), Symptom Normalization conceptual domain scores (*p* = 0.092), or Care-Seeking Delay conceptual domain scores (*p* = 0.247).

**Table 4:** Association of Participant Characteristics with GerdQ, GERD-HRQoL, and prespecified conceptual domain scores.

Variable	Group	GERD-Q Median (IQR) (*P* value)	GERD-HRQoL Median (IQR) (*P* value)	Symptom Normalization Conceptual Domain Score Median (IQR) (*P* value)	Care-Seeking Delay Conceptual Domain Score Median (IQR) (*P* value)
Age	18–40 years	5 (8)	7 (10)	11 (7)	12 (6)
	41–50 years	5 (7)	7 (9)	12 (10)	13.5 (7.75)
	51–60 years	7 (6)	11 (8)	13 (6)	12 (7)
	More than 60 years	5.5 (6.75) (*P* = 0.118)	8 (6.5) (*P* = 0.044)	11.5 (7) (*P* = 0.515)	12 (9) (*P* = 0.505)
	Male	5 (7)	8 (12)	12 (4)	13 (7)
	Female	5 (7) (*P* = 0.533)	7 (9) (*P* = 0.813)	12 (7) (*P* = 0.466)	12 (8) (*P* = 0.515)
	Primary	14 (-)	22 (-)	18 (0)	18 (-)
	Secondary	5 (7)	8 (9.5)	12 (6.5)	12 (7)
	Graduate	5.5 (8)	7 (9)	11 (8)	11 (7.75)
	Postgraduate	5 (9) (*P* = 0.183)	7 (11) (*P* = 0.256)	12 (3) (*P* = 0.054)	14 (4) (*P* = 0.010)
BMI	Underweight	8 (8)	9 (9)	12 (7)	13 (4.5)
	Normal	5 (7)	7 (8)	12 (7)	12 (7)
	Overweight	6 (7)	7 (11)	11 (9)	12 (8)
	Obese	2.5 (11) (*P* = 0.459)	7.5 (7) (*P* = 0.895)	12 (5) (*P* = 0.476)	13 (7.25) (*P* = 0.612)
Smoking	No	5 (7)	7 (9)	12 (7)	12 (8)
	Yes	5 (9) (*P* = 0.667)	10 (14) (*P* = 0.409)	13 (6) (*P* = 0.390)	14 (2) (*P* = 0.067)
Monthly Family Income	<5,000 SAR	5 (7)	8 (7)	12 (6)	12 (7)
	5,000–9,999 SAR	5.5 (7.75)	7 (9.75)	10 (10)	12 (8.5)
	10,000–14,999 SAR	6 (5.5)	8.5 (9.25)	12 (6)	12 (7)
	≥15,000 SAR	4 (8.5) (*P* = 0.141)	6 (9.5) (*P* = 0.293)	11 (7) (*P* = 0.259)	11 (7.5) (*P* = 0.523)
Residence	Rural	6 (5)	8 (9.5)	13 (4)	13 (3.5)
	City	5 (7) (*P* = 0.695)	7 (9) (*P* = 0.475)	11 (7) (*P* = 0.092)	12 (8) (*P* = 0.247)

Values are presented as median (IQR). GerdQ, GERD-Q; GERD-HRQoL, gastroesophageal reflux disease health-related quality of life. Mann–Whitney U test was used for comparisons between two groups (sex, smoking, and residence), and the Kruskal–Wallis test was used for comparisons among more than two groups (age, education, BMI, and monthly family income). *P* values <0.05 were considered statistically significant. NA: Interquartile range (IQR) could not be calculated because only two participants belonged to the primary education category.

Table 5 presents the Spearman correlation coefficients among GerdQ, GERD-HRQoL, and the prespecified Symptom Normalization and Care-Seeking Delay conceptual domain scores. GerdQ scores showed a strong positive correlation with GERD-HRQoL scores (*ρ* = 0.704, *P* < 0.001), indicating that greater GERD symptom severity was associated with poorer health-related quality of life. GerdQ scores also demonstrated weak but statistically significant positive correlations with the Symptom Normalization conceptual domain score (*ρ* = 0.165, *P* = 0.015) and the Care-Seeking Delay conceptual domain score (*ρ* = 0.166, *P* = 0.014). No significant correlations were observed between GERD-HRQoL and the Symptom Normalization conceptual domain score (*ρ* = 0.123, *P* = 0.071) or the Care-Seeking Delay conceptual domain score (*ρ* = 0.092, *P* = 0.175). In contrast, the Symptom Normalization conceptual domain score was strongly and positively correlated with the Care-Seeking Delay conceptual domain score (*ρ* = 0.734, *P* < 0.001), indicating that participants who were more likely to normalize their GERD symptoms were also more likely to delay seeking professional medical care.

**Table 5:** Spearman correlation matrix of GERD-Q, GERD-HRQoL, and the prespecified conceptual domain scores.

Variable	GerdQ	GERD-HRQoL	Symptom Normalization Conceptual Domain Score	Care-Seeking Delay Conceptual Domain Score
GerdQ	1	0.704 (<0.001)	0.165 (0.015)	0.166 (0.014)
GERD-HRQoL		1	0.123 (0.071)	0.092 (0.175)
Symptom Normalization Conceptual Domain Score			1	0.734 (<0.001)
Care-Seeking Delay Conceptual Domain Score				1

GERD-Q, Gastroesophageal Reflux Disease Questionnaire; GERD-HRQoL, Gastroesophageal Reflux Disease Health-Related Quality of Life Questionnaire. Values represent Spearman's rank correlation coefficients (*ρ*); *p*-values are shown in parentheses. Statistical significance was set at *p* < 0.05.

Table 6 presents the multivariable linear regression analysis examining factors associated with GERD-related health-related quality of life (GERD-HRQoL). The overall regression model was statistically significant [F(14, 203) = 14.875, *p* < 0.001] and explained 50.6% of the variance in GERD-HRQoL scores (R^2^ = 0.506; adjusted R^2^ = 0.472). Among the variables included in the model, GerdQ score was the only variable showing a statistically significant association with GERD-HRQoL. Higher GerdQ scores were associated with higher GERD-HRQoL scores (B = 0.971, 95% CI: 0.808–1.134, *p* < 0.001), indicating that greater current symptom burden was associated with poorer GERD-related health-related quality of life.

**Table 6:** Multivariable linear regression analysis of factors associated with GERD-related health-related quality of life.

Variables	B (Unstandardized coefficient)	Robust Standard Error	95% CI for B	*p*-value	VIF
Age group					
18–40 years	Reference	-	-	-	-
41–50 years	−0.374	1.055	−2.441 to 1.692	0.723	1.270
51–60 years	0.837	0.994	−1.111 to 2.785	0.400	1.178
Above 60 years	−0.444	0.662	−1.741 to 0.854	0.503	1.157
Gender					
Male	Reference	-	-	-	-
Female	−1.090	0.840	−2.736 to 0.557	0.195	1.121
Education					
Graduate	Reference	-	-	-	-
Primary	6.156	5.325	−4.281 to 16.594	0.248	1.130
Secondary	0.282	0.715	−1.119 to 1.684	0.693	1.094
Postgraduate	−0.573	1.206	−2.937 to 1.792	0.635	1.154
BMI					
Normal	Reference	-	-	-	-
Underweight	−0.169	1.013	−2.155 to 1.816	0.867	1.087
Overweight	−0.160	0.792	−1.713 to 1.393	0.840	1.168
Obese	1.209	1.055	−0.858 to 3.276	0.252	1.120
Smoking					
No	Reference	-	-	-	-
Yes	0.545	1.053	−1.519 to 2.609	0.605	1.171
GERD-Q Score	0.971	0.083	0.808 to 1.134	<0.001	1.090
Symptom Normalization Conceptual Domain Score	0.027	0.118	−0.205 to 0.259	0.819	2.572
Care-Seeking Delay Conceptual Domain Score	−0.076	0.118	−0.306 to 0.155	0.520	2.615
Model statistics					
R^2^	0.506				
Adjusted R^2^	0.472				
F-value (df)	14.875 (14, 203)				

B, unstandardized regression coefficient; robust SE, heteroscedasticity-robust standard error; CI, confidence interval; VIF, variance inflation factor. Reference categories were age 18–40 years, male gender, graduate education, normal BMI, and non-smoker. Age, gender, education, BMI, and smoking were entered using dummy coding. GERD-Q was entered as a continuous score. The prespecified Symptom Normalization and Care-Seeking Delay conceptual domain scores were entered as continuous variables, each calculated by summing its respective four items (possible range: 4–20), with higher scores indicating greater symptom normalization and greater care-seeking delay, respectively. Evidence of heteroscedasticity was identified during residual diagnostics; therefore, robust covariance estimates were used for standard errors, 95% confidence intervals, and *p*-values. VIF and model-fit statistics were obtained from the corresponding ordinary least-squares model. Dependent variable: GERD-HRQoL score. Multicollinearity was assessed using the variance inflation factor (VIF), with values ranging from 1.087 to 2.615.

None of the demographic or clinical variables, including age group, gender, educational level, BMI category, and smoking status, showed a statistically significant association with GERD-HRQoL after adjustment for the other variables in the model. Similarly, the Symptom Normalization conceptual domain score (B = 0.027, 95% CI: −0.205 to 0.259, *p* = 0.819) and the Care-Seeking Delay conceptual domain score (B = −0.076, 95% CI: −0.306 to 0.155, *p* = 0.520) were not independently associated with GERD-HRQoL after adjustment for the other variables. VIF values ranged from 1.087 to 2.615, indicating no evidence of problematic multicollinearity among the variables. Figure 1 shows the adjusted regression coefficients from the multivariable regression model. GerdQ score was the only factor significantly associated with GERD-related health-related quality of life (GERD-HRQoL) after adjustment. Age, sex, educational level, BMI, smoking status, the Symptom Normalization conceptual domain score, and the Care-Seeking Delay conceptual domain score were not significantly associated with GERD-HRQoL.

**Figure 1:**
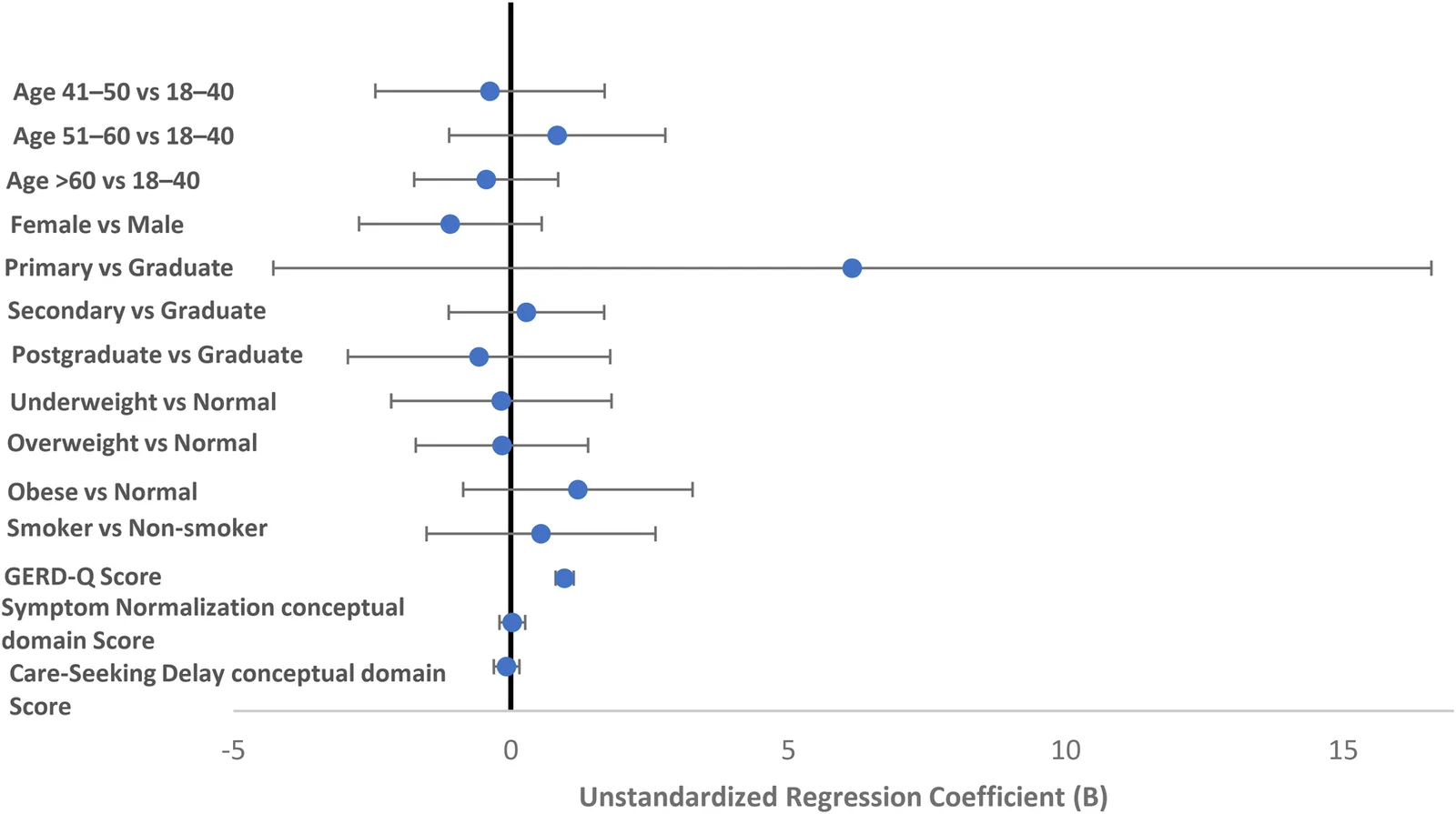
Forest plot of factors associated with GERD-related health-related quality of life in the multivariable regression model. The forest plot presents the unstandardized regression coefficients (B) and robust 95% confidence intervals for factors associated with GERD-related health-related quality of life (GERD-HRQoL). The vertical line at B = 0 represents the null value. Reference categories were age 18–40 years, male sex, graduate education, normal BMI, and non-smoking status.

## 4 Discussion

The findings showed that greater GERD symptom severity was associated with poorer GERD-related quality of life. Symptom normalization demonstrated a strong positive correlation with delayed healthcare-seeking behaviour, although neither the Symptom Normalization conceptual domain score nor the Care-Seeking Delay conceptual domain score independently predicted GERD-related quality of life after multivariable adjustment. Participants were predominantly female (87.6%), younger adults aged 18–40 years (49.0%), and highly educated, with 65.6% having graduate or postgraduate qualifications. Similar demographic characteristics have been reported in previous Saudi studies, where females comprised 69.9% of participants and 71.1% had university-level education (34). Likewise, most participants in our study resided in urban areas (92.2%), consistent with the demographic profile of earlier Saudi studies (34, 35). In the present study, 28.0% of participants were classified as overweight and 8.3% as obese. Although previous studies have suggested that excess body weight may contribute to GERD through mechanisms such as increased intra-abdominal pressure and alterations in gastroesophageal junction function (36), BMI was not significantly associated with GERD symptom burden or GERD-related quality of life in the present study. This discrepancy may be related to differences in study population characteristics, BMI distribution, or other factors influencing GERD symptom expression. Similar to previous reports, the predominance of female participants may reflect differences in symptom perception and reporting between women and men (37, 38).

Although age was not significantly associated with GERD symptom burden (*p* = 0.118), older participants (>60 years) had significantly poorer GERD-related quality of life than younger age groups (*p* = 0.044). This suggests that older adults in this cohort experienced greater GERD-related quality-of-life impairment despite similar symptom severity, although the cross-sectional design prevents determination of temporal or causal relationships. Similar findings have been reported in previous studies, although variations across studies may reflect differences in population characteristics, lifestyle factors, comorbidities, and symptom perception (39). The predominance of female participants may reflect greater healthcare utilization or symptom reporting among women rather than a true difference in GERD prevalence. Previous studies have reported variable associations between sex and GERD, with some showing similar prevalence between men and women, while others have observed differences in symptom perception and quality-of-life impact (40).

Interestingly, educational level was associated with the Care-Seeking Delay conceptual domain score, with participants having postgraduate education reporting higher delay scores. This finding may reflect differences in symptom appraisal, self-management practices, or perceptions regarding the need for professional care; however, these factors were not assessed in the present study and require further investigation. Greater GERD symptom severity was associated with poorer GERD-related quality of life, consistent with previous studies demonstrating that GERD substantially affects patients’ daily functioning and quality of life (31, 41, 42). Previous studies have reported that GERD is associated with impaired physical activity, social functioning, sleep, and work productivity (43–45), with greater symptom severity consistently associated with poorer health-related quality of life (46–53). More than half of the participants reported modifying their diet because of GERD symptoms, indicating that dietary adjustment is a common behavioural response among individuals attempting to manage reflux-related symptoms. Similar findings have been reported in a previous study, where patients frequently modified their dietary habits or avoided foods perceived to aggravate reflux symptoms as part of symptom management (34). In addition, 43.6% reported heartburn-related sleep impairment on the GERD-HRQoL scale, while 57.3% reported GERD-related sleep disturbance during the previous week according to GerdQ responses. These findings are consistent with previous studies reporting associations between GERD symptoms and sleep disturbance, reduced concentration, limitations in physical activity, and lower work productivity (54–58).

The correlation analysis demonstrated a strong positive association between GERD symptom severity and GERD-related quality of life, indicating that higher symptom burden was associated with greater impairment in disease-specific quality of life. This finding is consistent with previous studies showing that symptom severity is a major determinant of reduced health-related quality of life among patients with GERD (31, 59–61). The Symptom Normalization and Care-Seeking Delay conceptual domain scores showed weak but statistically significant positive correlations with GERD symptom severity, whereas the strongest association between the two conceptual domain scores was observed between symptom normalization and care-seeking delay. Participants who reported greater symptom normalization also tended to report greater delay in seeking professional medical care. Similar observations have been reported in population-based and qualitative studies, where individuals with reflux symptoms frequently relied on self-management, perceived their symptoms as mild, or did not seek medical care because of their beliefs about symptom seriousness or treatment (62, 63). The relatively strong correlation between the Symptom Normalization and Care-Seeking Delay conceptual domain scores (*ρ* = 0.734), together with the one-factor structure identified by exploratory factor analysis, suggests substantial overlap between these prespecified conceptual domains. This may indicate that symptom normalization and delayed care-seeking represent closely related aspects of a broader behavioral process rather than psychometrically distinct constructs. However, the two domains were prespecified conceptually to capture different aspects of patient behavior and were therefore retained as separate domain scores. Further studies using confirmatory factor analysis and discriminant-validity testing are needed to clarify whether these domains represent distinct constructs or a common underlying behavioral dimension. Collectively, these findings highlight the potential importance of improving awareness of GERD symptoms and healthcare-seeking behaviour. However, because of the cross-sectional design, the temporal relationship between symptom normalization and delayed healthcare-seeking cannot be determined.

The strong association between GerdQ and GERD-HRQoL should be interpreted in light of the conceptual overlap between the two instruments. Both measures assess aspects of GERD symptom burden, including heartburn, regurgitation, sleep disturbance, medication use, and symptom frequency or severity (32, 33, 64). Therefore, the observed association may partly reflect shared symptom-related content between the instruments. In contrast, the purpose-designed conceptual domain scores assessed symptom normalization and care-seeking delay, representing behavioral dimensions beyond current symptom severity. In the present study, these domain scores were strongly correlated with each other but showed only weak correlations with GerdQ and no significant correlations with GERD-HRQoL. Furthermore, neither conceptual domain score was independently associated with GERD-HRQoL after adjustment for GerdQ and other covariates, indicating that their independent contribution to GERD-related quality of life was not demonstrated in this study.

In the multivariable regression analysis, GERD symptom severity was the only variable independently associated with GERD-related quality-of-life impairment. Participants with higher GerdQ scores had poorer GERD-related quality of life after adjustment for demographic characteristics, lifestyle factors, the prespecified conceptual domain scores for symptom normalization and care-seeking delay, and other covariates. This finding is consistent with previous studies demonstrating that increasing reflux symptom severity is strongly associated with impaired disease-specific quality of life and poorer physical and psychological well-being (59). In contrast, age, gender, BMI, smoking status, the Symptom Normalization conceptual domain score, and the Care-Seeking Delay conceptual domain score were not independently associated with GERD-related quality of life after adjustment. Although the two conceptual domain scores were weakly but significantly correlated with GERD symptom severity, neither independently predicted GERD-related quality of life in the multivariable analysis, suggesting that their association with quality of life may be explained partly by their relationship with symptom severity. Likewise, previous studies have reported inconsistent associations between demographic characteristics, particularly sex, and GERD-related quality of life (59, 65–67), which is consistent with the present findings.

### 4.1 Clinical implications

The findings highlight the importance of routinely assessing GERD symptom severity in clinical practice, as symptom burden was the strongest factor associated with GERD-related health-related quality of life. Healthcare professionals should also be aware that some patients may normalize reflux symptoms and delay seeking medical care, which may be relevant when assessing patients’ symptom perception and healthcare-seeking patterns. Patient education focused on improving symptom recognition and promoting awareness of timely healthcare-seeking may be considered as part of GERD management. Longitudinal and interventional research is warranted to evaluate whether approaches addressing symptom normalization and healthcare-seeking behaviour are associated with patient outcomes.

### 4.2 Limitations

This study has several limitations. First, the cross-sectional design precludes the establishment of temporal or causal relationships between GERD symptom severity, symptom normalization, care-seeking delay, and health-related quality of life. Second, participants were recruited consecutively from the outpatient clinics of King Khalid University Medical City (KKUMC) and affiliated primary healthcare centers within a single region of Saudi Arabia, which may limit the generalizability of the findings to other populations and healthcare settings. In addition, the sample was predominantly female, relatively young-to-middle-aged, highly educated, and mostly non-smoking, which may limit the applicability of the findings to men, older adults, rural populations, smokers, and other patient groups with different sociodemographic or clinical characteristics. Therefore, the findings should be interpreted primarily in the context of this clinic-based population in the Aseer region of Saudi Arabia rather than generalized to the broader Saudi population. Third, the use of self-reported questionnaires may have introduced recall and reporting bias, despite research staff being available to clarify questionnaire items when required. In addition, the sample size was determined based on the feasibility of recruitment during the study period rather than an *a priori* statistical power or precision calculation. Therefore, statistical power was not prospectively determined, and limited power for some analyses cannot be excluded. Fourth, medication and treatment-related variables were not collected. Specifically, information on proton pump inhibitor use, treatment adherence, over-the-counter antacid use, prior endoscopy, duration of GERD, and current treatment status was unavailable. These factors may be relevant to symptom burden, symptom normalization, care-seeking behavior, and health-related quality of life, and their potential contribution to the observed associations could therefore not be evaluated. The absence of treatment-status information may also partly explain the relatively low proportion of participants with a current GerdQ score ≥8 despite physician-established GERD, as some participants may have had controlled or treated disease at the time of assessment. Consequently, residual confounding from unmeasured treatment-related and other clinical factors cannot be excluded. Finally, the purpose-designed items demonstrated good internal consistency and supported a common underlying factor; however, the EFA did not establish discriminant validity between the two prespecified conceptual domains. Further validation in independent and more diverse populations, including confirmatory factor analysis and discriminant-validity assessment, is warranted.

## 5 Conclusion

This cross-sectional study found that symptom normalization and delayed healthcare-seeking behaviors were commonly reported among adults with physician-diagnosed GERD. GerdQ scores were used to characterize current symptom burden rather than to establish or exclude the diagnosis of GERD. Greater GERD symptom burden was strongly associated with poorer GERD-specific health-related quality of life and was the only variable significantly associated with GERD-HRQoL in the multivariable regression analysis. The prespecified Symptom Normalization and Care-Seeking Delay conceptual domain scores were strongly positively correlated with each other; however, neither conceptual domain score was independently associated with GERD-HRQoL after adjustment. Overall, the findings indicate a strong association between symptom normalization and care-seeking delay, whereas the adjusted association with GERD-HRQoL was observed for symptom burden. Future longitudinal and multicenter studies involving diverse populations are warranted to further examine the temporal relationships among symptom appraisal, care-seeking behavior, symptom burden, and quality-of-life outcomes.

## Data Availability

The original contributions presented in the study are included in the article/Supplementary Material, further inquiries can be directed to the corresponding author.
