# Supplementary material for: Symptom normalization, care-seeking delay, and health-related quality of life in adults with gastroesophageal reflux disease: a cross-sectional study
Source: Front Med (Lausanne). 2026 Oct 14;13:1969209. doi: 10.3389/fmed.2026.1969209 (PMC13713310; doi:10.3389/fmed.2026.1969209)
Supplement: Supplementary file 1 [file Supplementaryfile1.docx]

**Table 1. Summary Statistics and Reliability of Study Questionnaires**

| **Questionnaire** | **No. of Items** | **Possible Score Range** | **Mean ± SD** | **Median (IQR)** | **Min–Max** | **Cronbach's α** |
| --- | --- | --- | --- | --- | --- | --- |
| GERD-Q | 6 | 0–18 | 5.78 ± 4.59 | 5 (7) | 0–18 | 0.85 |
| GERD-HRQoL | 10 | 0–50 | 8.93 ± 6.48 | 7 (9) | 0–32 | 0.76 |
| Symptom Normalization | 4 | 4-20 | 10.50 ± 4.34 | 12 (7) | 4-20 | 0.866 |
| Care-Seeking Delay | 4 | 4-20 | 10.95 ± 4.45 | 12 (8) | 4-20 | 0.914 |

**Table 2. Exploratory Factor Analysis of the Symptom Normalization and Care-Seeking Delay Questionnaire**

| **Model Fit** | | | |
| --- | --- | --- | --- |
| **Parameter** | | **Result** | |
| **KMO Measure** | | 0.901 | |
| **Bartlett's χ²** | | 1333.614 | |
| **Degrees of Freedom** | | 28 | |
| **p-value** | | <0.001 | |
| **Extraction Method** | | **Principal Axis Factoring** | |
| **Rotation Method** | | **None (one-factor solution)** | |
| **Factors Retained** | | 1 | |
| **Initial Eigenvalue** | | 5.332 | |
| **Variance Explained (%)** | | 66.65 | |
| **Extracted Variance (%)** | | 62.18 | |
| **Factor Matrix** | | | |
| **Item** | **Questionnaire item** | **Factor 1** |  |
| **Care-Seeking Delay 1** | It took me a long time to seek professional care after my symptoms began. | 0.733 |  |
| **Care-Seeking Delay 2** | I delayed seeing a doctor because I thought the symptoms might go away. | 0.872 |  |
| **Care-Seeking Delay 3** | I postponed medical care because I wasn’t sure my symptoms were serious. | 0.830 |  |
| **Care-Seeking Delay 4** | I delayed medical care because I expected the symptoms to improve on their own. | 0.858 |  |
| **Symptom Normalization 1** | My reflux symptoms seem like a normal part of life. | 0.602 |  |
| **Symptom Normalization 2** | I have often ignored my GERD symptoms instead of seeking medical help. | 0.795 |  |
| **Symptom Normalization 3** | I think my symptoms are not serious enough to see a doctor. | 0.804 |  |
| **Symptom Normalization 4** | I usually manage my symptoms on my own without medical help. | 0.782 |  |

**Note:** Items were rated on a 5-point Likert scale (1 = strongly disagree to 5 = strongly agree), with neutral responses scored as 3; no items were reverse scored. Symptom normalization and care-seeking delay were each scored by summing their respective four items (possible range: 4–20), with higher scores indicating greater symptom normalization and greater care-seeking delay, respectively. Principal Axis Factoring was used for extraction. Factor retention was evaluated using eigenvalues, scree-plot inspection, and parallel analysis; parallel analysis supported retention of one factor. Accordingly, no rotation was applied. All eight items had factor loadings ≥0.60.

**Table 3. Assessment of Normality for Continuous Study Variables**

| **Variable** | **Kolmogorov–Smirnov Statistic** | **p-value** | **Shapiro–Wilk Statistic** | **p-value** | **Distribution** |
| --- | --- | --- | --- | --- | --- |
| GERD-Q Score | 0.108 | <0.001 | 0.938 | <0.001 | Non-normal |
| GERD-HRQoL Score | 0.135 | <0.001 | 0.924 | <0.001 | Non-normal |
| Symptom Normalization Score | 0.149 | <0.001 | 0.915 | <0.001 | Non-normal |
| Care-Seeking Delay Score | 0.167 | <0.001 | 0.924 | <0.001 | Non-normal |
